# Structure and function of vimentin in the generation and secretion of extracellular vimentin in response to inflammation

**DOI:** 10.1186/s12964-025-02194-z

**Published:** 2025-04-18

**Authors:** Zhiyao Yuan, Paul A. Janmey, Christopher A. McCulloch

**Affiliations:** 1 Nanjing Stomatological Hospital, Affiliated Hospital of Medical School, Research Institute of Stomatology, Nanjing University, Nanjing, China; 2 Dept. of Physiology, University of Pennsylvania, Philadelphia, PA 19104 USA; 3 Faculty of Dentistry, University of Toronto, Room 461, 124 Edward Street, Toronto, ON M5G 1G6 Canada

**Keywords:** Vimentin, Extracellular vimentin (ECV), Inflammation, Post-translational modifications (PTMs), Unconventional protein secretion (UPS)

## Abstract

The canonical functions of vimentin in cell mechanics and migration have been recently expanded by the discovery of new roles for extracellular vimentin (ECV) in immune responses to infection, injury and cancer. In contrast with the predominantly filamentous form of intracellular vimentin, ECV exists largely as soluble oligomers. The release of ECV from intact cells is dependent on mechanisms that regulate the assembly and disassembly of intracellular vimentin, which are influenced by discrete post-translational modifications. In this review we highlight the processes that promote the conversion of intracellular and insoluble vimentin filaments to ECV and secretion mechanisms. Insights into the regulation of ECV release from stromal and immune cells could provide new diagnostic and therapeutic approaches for assessing and controlling inflammatory diseases.

## Introduction

Vimentin, an intermediate filament cytoskeletal protein, assembles into mechanically stable intracellular filaments in a hierarchical manner to protect cells from mechanical stress. However, in certain inflammatory lesions, vimentin is also found outside cells as extracellular vimentin (ECV), which is thought to impact local immune responses. In cells located in inflamed tissues, intracellular vimentin filaments undergo disassembly into soluble forms (small oligomers), a process that is dependent in part on post-translational modifications (PTMs) [1].While the mechanisms that enable ECV to enter the extracellular space are not well defined, a recent paper has indicated that ECV may be released through the type III unconventional protein secretion (UPS) system [2]. ECV has been detected on the outer surface of exosomes after release [3, 4]. Evidently, ECV can play a pro-inflammatory role in tissues but it can also inhibit the immune response to cancer cells [5] and promote wound healing [6]. In this review, we examine three key processes essential for the release of ECV: the conversion of intracellular vimentin filaments to soluble forms in response to inflammation; the increase in vimentin solubility mediated by PTMs; and the export mechanisms which mediate the entry of soluble vimentin into the extracellular space.

## Vimentin- a type III intermediate filament protein

Intermediate filaments (IF) comprise a family of cytoskeletal proteins that provide structural support and mechanical stability to cells, facilitate organelle and cargo transport, and contribute to cell adhesion and migration [7–9]. Based on their amino acid sequences, IF proteins are classified into six different types, which are often discretely expressed in specific cell types and tissues. Type I (acidic keratins) and type II (neutral/basic keratins) are primarily expressed in epithelial cells and are the major constituents of hair and nails. Type IV (neurofilament proteins) are the major IFs of neurons, Type VI proteins (e.g., nestin) are expressed in neuronal progenitor cells of the central nervous system, and Type V (nuclear lamins) are enriched in the nuclear envelope. Type III IF proteins are expressed in diverse cell types (e.g., desmin in muscle cells, glial fibrillary acidic protein in glial cells, and peripherin in certain cells of the peripheral nervous system). Here we focus on vimentin, a 54 kDa type III IF protein that is broadly expressed by cells of mesenchymal origin, as well as by certain types of cancer cells undergoing epithelial-mesenchymal transition.

The English word vimentin is derived from the Latin word “vimentum”, which originally referred to an array of flexible willow rods used for basket making. In human cells vimentin is encoded by the *VIM* gene, which evolved from a common ancestor of other IF proteins [10, 11]. Vimentin may have emerged prior to other type III IF genes and is expressed in mesodermal and neural precursor cells in early stages of embryogenesis [12]. Following its initial expression in these cell types, vimentin is often downregulated, which is followed by the accumulation of other, distinct IF proteins [13]. While vimentin filaments are broadly expressed in different cell lineages during embryonic development, in postnatal life vimentin expression is limited and is primarily restricted to specific cell types including for example, fibroblasts, endothelial cells, muscle cells, immune cells, and other tissue cells but particularly those of mesodermal origin [2, 14–19].

### Physiological functions of vimentin

Intracellular vimentin is involved in a broad array of cellular processes including proliferation, cell division, adhesion, and migration [20–22], all of which are important for cell responses to injury, including wound repair [23]. Vimentin filaments contribute to the mechanical stability of cells and to the integrity of the nucleus in particular [24]. In adult tissues vimentin is commonly considered as a marker of mesenchymal lineages [25]. In addition, vimentin expression is used to assess epithelial-mesenchymal transition (EMT), a process in which cells transform from an epithelial into a mesenchymal phenotype. Some of the diverse biological associations of vimentin expression in healthy and inflamed tissues are listed in Table 1.

**Table 1:** Physiological functions of intracellular vimentin

Physiological Functions of Vimentin	References
mechanical protection;	[20]
scaffold for other proteins;	[26]
organelle anchoring;	[27]
cell proliferation;	[21]
cell division; cell adhesion; cell migration;	[22]
cell signaling;	[28]
osteoblast differentiation inhibition;	[29]
allergy	[30]
NETs formation	[17]

### Role of Vimentin in Pathological Processes

Studies of mice with genetic deletion of vimentin show that these animals reach adulthood uneventfully and exhibit normal reproductive capability [23], but they exhibit an array of distinct abnormalities when challenged  , including alterations of repair in response to tissue injury (Table 2). Deficiency of vimentin in mice impairs fibroblast migration and matrix contractile capacity, delays the transformation of fibroblasts into myofibroblasts, and diminishes the integrity of cells and tissues. All of these features contribute to reduced wound closure and as a result, dysfunctional wound healing [31]. These data are consistent with cell culture studies indicating that vimentin filaments affect cell mechanics and are involved in signal transduction, mechanosensing, cell migration and notably, the inflammatory response [23]. Vimentin contributes to the inflammatory response by regulating, for example, the activation of the NLRP3 inflammasome [32] and the maturation of IL-1β. In vimentin KO mice exposed to lipopolysaccharide, asbestos, or bleomycin, there are reduced levels of mature IL-1β and consequently, lung alveolar damage, inflammatory cell infiltration, and pulmonary fibrosis are diminished [32].

Vimentin deficiency inhibits the development of atherosclerotic plaques in mouse models of atherosclerosis, this inhibition is attributed in part to an enhanced capacity of mouse peritoneal macrophages to internalize oxidized low-density lipoprotein (oxLDL), which diminishes the formation of foam cells, a pivotal early stage in the pathogenesis of atherosclerosis [33]. Vimentin is highly expressed in fiber cells of the eye lens and plays a crucial role in maintaining lens integrity and clear vision. Abnormalities in vimentin filament assembly may also affect the function of the lens, an aberration which is associated with the formation of cataracts [34]. Further, and as indicated above, vimentin regulates EMT, which can thereby affect cancer metastasis [35]. Collectively, these data underline the multiple roles of vimentin in the pathogenesis of diverse diseases, including inflammatory disorders.

**Table 2:** Role of vimentin in pathological processes

Roles of Vimentin in Pathological Process	Involvement in Pathogenic Mechanisms		Examples of Related Diseases
-Chronic inflammation, -Fibrosis [32]		Activation of the NLRP3 inflammasome and the maturation of IL-1β	Pulmonary fibrosis
-Dysfunctional wound Healing [31]		Fibroblast migration and transformation into myofibroblasts	Keloid formation
-Formation of foam cells [33]		Facilitate macrophages to internalize oxidized low-density lipoprotein (oxLDL)	Atherosclerosis [19]
- Dysfunction of the lens [34]		Maintaining lens integrity	Cataracts [34]
-Cancer metastasis [35]		Regulates EMT	Cancers
-Viral and bacterial infection [36, 37]		Act as adhesive co-receptor for pathogen; Form ‘vimentin cage’ to facilitate pathogen replication [38]	SARS [39] Encephalitis [40]

### Regulation of vimentin expression

The pathological processes described above are often associated with increased or decreased intracellular vimentin compared with healthy cells, suggesting that vimentin expression levels may be a determinant of ECV abundance in cells found in specific inflammatory lesions. The human *VIM* gene is located on chromosome 10p13; its promoter sequence (~ 2000 bp) is predicted to bind up to 79 transcription factors [41, 42] (e.g., NF-κB [43], PEA3 [44], Sp1, ZBP-89 [45]), suggesting complex regulatory processes. As genetic regulation encompasses epigenetic mechanisms such as methylation [46] and histone modifications [47], we note that inflammation can alter epigenetic modifications [48], which may in turn affect vimentin expression in inflamed lesions. Further, vimentin expression can be post-transcriptionally regulated by various non-coding RNAs including microRNA-30a, circular RNA circ-10,720, and long non-coding RNA GAS5 [49–51]. The function of these non-coding RNAs is affected at inflamed sites [52, 53], which may thereby alter vimentin expression and subsequently the amount of released ECV. In addition, vimentin expression is regulated by PTMs such as ubiquitination [54], which may influence the stability or disassembly of vimentin filaments within the cell. Several other types of PTMs [55] discussed below, can affect the disassembly of cytoskeletal vimentin and the release of ECV. In inflammation, the activation of certain signaling pathways (e.g., NF-κB) can affect the activity of ubiquitin ligases [56], which modify vimentin ubiquitination and its stability and degradation through the ubiquitin-proteasome pathway.

Certain extracellular signals and cell states associated with inflammation can affect vimentin expression levels. For example, platelet-derived growth factor, a pro-fibrotic cytokine that promotes the proliferation and migration of fibroblasts at inflamed sites, and 12-O-tetradecanoylphorbol 13-acetate, a potent activator of protein kinase C, enhance vimentin expression [57]. Cells with enhanced invasive potential (e.g., cells undergoing EMT) exhibit downregulation of epithelial markers like E-cadherin and upregulation of mesenchymal markers, including vimentin, which affects their migratory and invasive behavior [58]. In senescent cells, vimentin expression is increased, leading to more highly abundant intermediate filament (IF) bundles. These structural modifications may alter cell motility and the mechanical properties of cells, potentially enhancing the susceptibility of aging cells to inflammation-induced dysfunction [14].

## Assembly of vimentin filaments: from soluble subunits to insoluble mature IFs

In cultured fibroblasts, bundled vimentin filaments extend from the nuclear membrane to the plasma membrane to provide structural support and enable intracellular trafficking of various cargoes. Vimentin filaments also form a cage-like network around the nucleus that protects the nucleus from deformation and DNA damage while also participating in mechanotransduction [24]. During cell spreading and cell adhesion to matrix, vimentin appears as dot-like structures or squiggles that extend to the cell periphery. These dot-like structures are likely precursors of new filaments [59, 60]. Under certain physiological or pathological conditions, vimentin filaments depolymerize, which increases their solubility and is associated with the more diffuse distribution of vimentin across the cytoplasm [61].

**Fig. 1:**
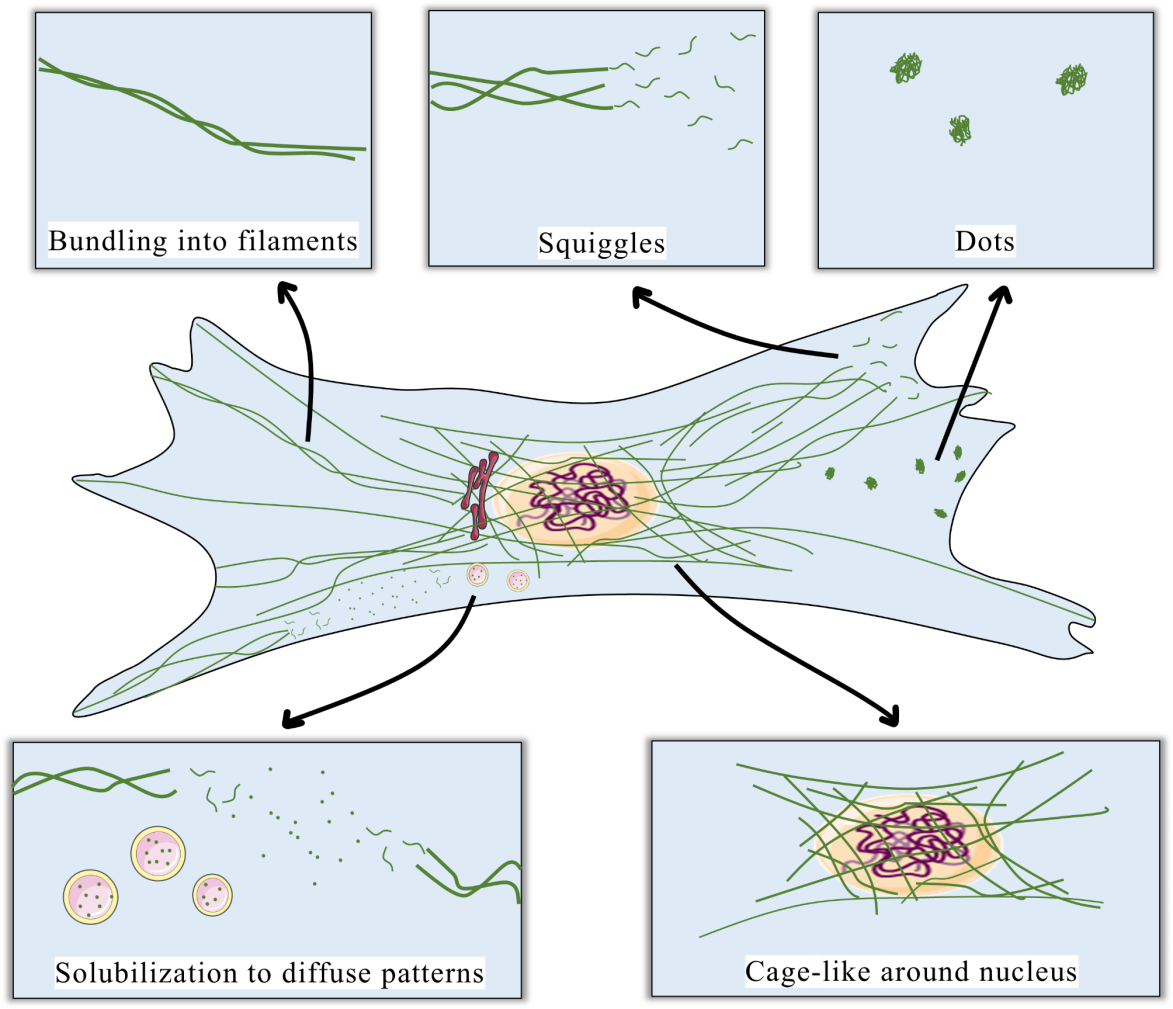
Dynamic conformational changes of vimentin. Typically, vimentin monomers assemble into filaments, which extend from the nuclear to the plasma membrane. Around the nucleus, vimentin filaments form a cage-like network. During cell spreading and adhesion, vimentin forms dot-like structures or squiggles that extend to the periphery of the cell and are considered as precursors of new filaments. Under certain conditions, increased vimentin solubility manifests as diffuse patterns. These localized pools of soluble vimentin could reassemble at distinct cellular locales and potentially be secreted outside the cell

Fluorescence recovery after photobleaching has been used to observe the movement of, and dynamic exchange between, vimentin subunits and filaments, processes that are involved in filament assembly at distinct sites [62]. These studies demonstrate the broad range of the filament configurations of vimentin in physiological conditions and how the dynamics of filament turnover contribute to the structural integrity of cells and influence cell responses to various stimuli [59, 60, 63] (Fig. 1).

When the assembly of vimentin filaments from subunits and filament turnover are perturbed by inflammatory conditions [17], the subunit/filament equilibrium shifts to favor a net increase of soluble vimentin inside the cell, some of which is secreted under perturbed conditions [1]. In the following sections of this review, we examine the structural features of vimentin that contribute to its intracellular stability and function. We also discuss how these insights can inform our understanding of the mechanisms underlying vimentin disassembly and its subsequent release from cells as ECV.

Unlike actin in which filament assembly can be initiated by the addition of monomers on to small actin seeds mediated through formins, vimentin filaments assemble in a hierarchical fashion, culminating in the formation of highly stable structures. In contrast to the polarity exhibited by microtubules and actin filaments, vimentin filaments are non-polar [64]. Recent data obtained from cryo-focused ion-beam milling, cryo-electron microscopy and tomography provided a three-dimensional structure of vimentin filaments [65]. This proposed structure suggests an arrangement of modular, intertwined helical structures, which in cross-section contain 40 α-helices. These helices are in turn organized into five protofibrils. The intrinsically disordered head domain concentrates in the lumen of filaments. The intrinsically disordered tail domain forms lateral connections between the protofibrils [65].

**Fig. 2:**
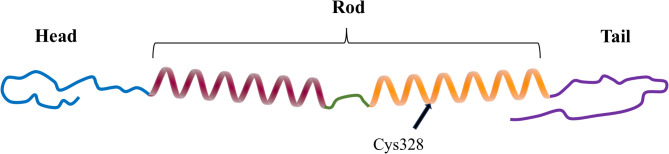
Structure of the vimentin molecule. Similar to other type III intermediate filaments, vimentin consists of a highly conserved central α-helical rod domain (312 amino acids) flanked by a head domain (95 amino acids) and a tail domain (59 amino acids). The head and tail domains are inherently disordered. (Information from Uniprot [66]). Vimentin undergoes various PTMs, including phosphorylation, glycosylation, and ADP-ribosylation in its head and tail domains, and glutathionylation of Cys328 in the rod domain (arrow). These modifications enable important regulatory mechanisms that dictate the assembly, disassembly, and overall functions of vimentin filaments

**Fig. 3:**
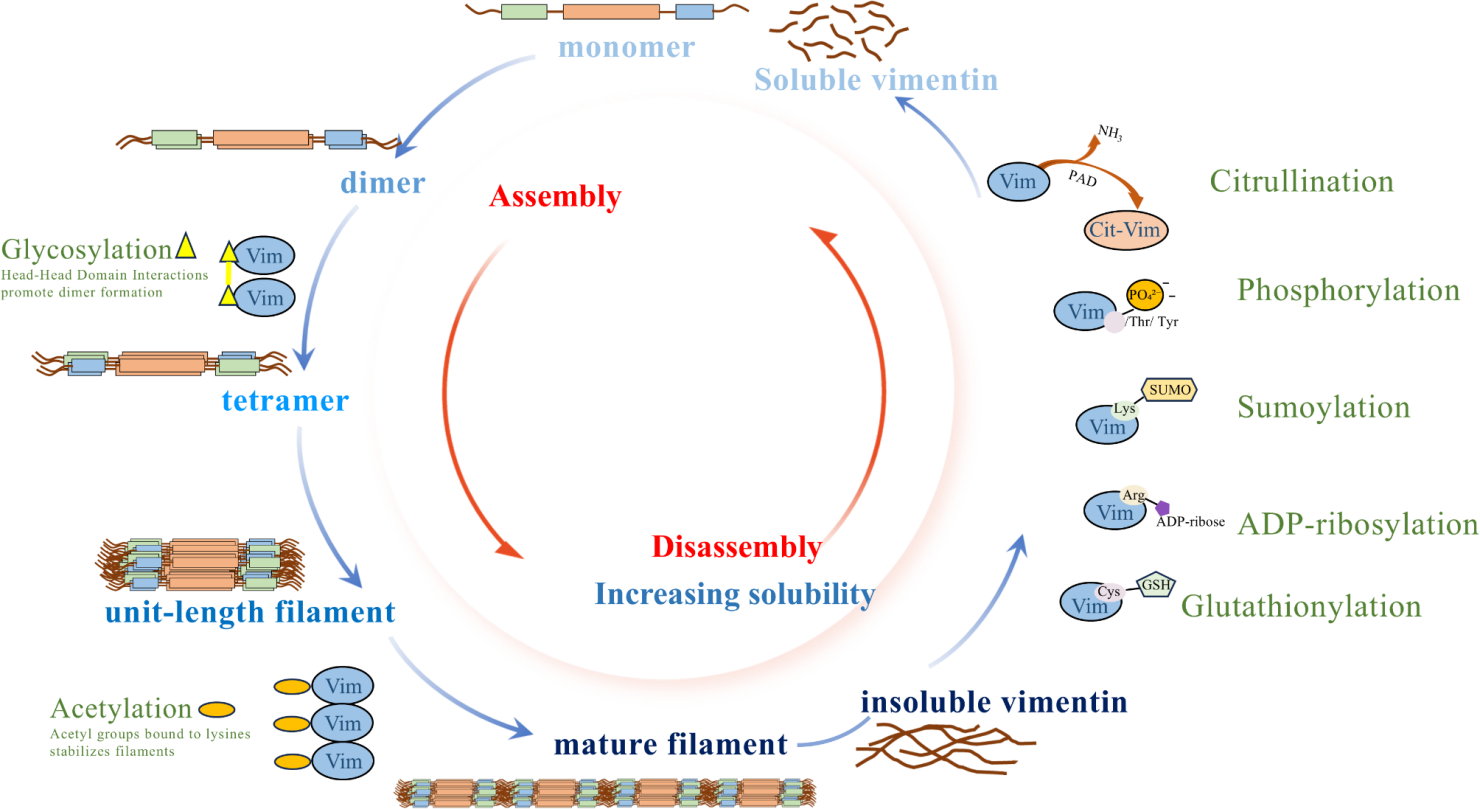
Assembly and disassembly of vimentin IFs. Vimentin monomers initially combine (top of figure) to form coiled-coil dimers that subsequently assemble into antiparallel tetramers. Eight soluble tetramers associate laterally, creating unit-length filaments (ULFs). By longitudinal fusion driven by ‘head-to-tail’ interactions, ULFs combine to form insoluble, mature vimentin filaments (diameter: 10–12 nm). Mature filaments can undergo disassembly into more soluble polymers, which depends on regulation by different post-translational modifications (PTMs). Assembly and disassembly are dynamic processes that enable adaptation to alterations in cellular physiological states and external environmental stressors. PTMs such as glycosylation and acetylation promote vimentin filament stability while other PTMs such as citrullination, phosphorylation, sumoylation, ADP-ribosylation and glutathionylation enhance filament disassembly and ultimately, the generation of more solution vimentin

Vimentin assembly commences with the formation of soluble oligomeric complexes. These complexes transition from monomers (Fig. 2) that combine into parallel coiled-coil dimers, which then assemble in an antiparallel manner into symmetric tetramers. Subsequently, eight tetramers rapidly (< 1 second) associate laterally to form unit-length filaments (ULFs), which can grow to form the filament network. Over a highly variable time course (1 second to 20 minutes) the longitudinal fusion of ULFs, which is driven by ‘head-to-tail’ interactions, leads to the formation of insoluble mature vimentin filaments [67] (Fig. 3). This process is regulated by conserved IF consensus motifs located at both ends of the central a-helical rod domain. The assembly process is thought to endow vimentin filaments with their characteristic flexibility and extensibility [68–70].

ECV, unlike typical intracellular vimentin filaments, mainly exists in the form of oligomers, about 4–12 monomers, which is smaller than a single ULF (8 tetramers). The oligomeric structures of vimentin have high affinity for lipid bilayers, which is in contrast to vimentin filaments. Accordingly, vimentin may more readily incorporate into the plasma membrane after transitioning from a typical filament to an oligomeric structure [71]. In vitro studies using purified vimentin show that vimentin assembly and disassembly can be modulated by adjusting the ionic strength of the incubating buffer [72]. In neutral buffers at low ionic concentrations, vimentin is maintained as a soluble tetramer. Additional increases of ionic strength will promote the assembly of vimentin into ULFs, which ultimately form filaments.

When considering these processes in intact cells, vimentin assembly and disassembly are dynamic processes that enable adaptations to external environmental stressors. These adaptations include several PTMs (see below) that influence the assembly and disassembly of IF proteins and cytoplasmic enzymes, such as calpain, which cleaves vimentin into smaller fragments, thereby potentially facilitating secretion [73]. In the context of inflammation, it is useful to consider the regulation of vimentin assembly and disassembly, since inflammatory lesions are often associated with increased vimentin secretion (see Sect. 6 below), which in turn is dependent on vimentin disassembly.

## Regulation of vimentin assembly and disassembly by PTMs

Vimentin filament assembly and disassembly are regulated by PTMs, which include phosphorylation, glutathionylation, sumoylation, glycosylation and citrullination (described in more detail below; see Fig. 3 above). These PTMs affect processes that enable the conversion of insoluble filaments into soluble complexes, which are then more likely to be secreted as ECV. Notably, the vimentin head domain is crucial for the stability of tetramers and filament assembly: molecules that lack the N-terminal domain fail to assemble into 10 nm filaments and remain soluble [69]. Conceivably in inflamed tissues, PTMs that occur in the vimentin head domain and that normally regulate filament assembly, could also contribute to increased vimentin solubility inside cells.

### Phosphorylation

One of the most extensively studied PTMs that regulate the assembly dynamics of vimentin filaments is phosphorylation [74], a modification that targets serine and threonine residues along with a smaller number of tyrosine residues. Vimentin filaments are phosphorylated at multiple sites by several kinases and this modification is associated with their dynamic assembly and disassembly [75]. Vimentin phosphorylation sites are primarily located in the vimentin head and tail domains. Phosphorylation sites located in the head domain can interfere with interactions between VIM dimers, which may inhibit the formation of VIM tetramers that are needed for the subsequent assembly into filaments. Residues in the rod domain are not as frequently phosphorylated, possibly due to the coiled structure that restricts the access of kinases [76].

During mitosis and in migrating cells, and potentially in inflammation, the level of phosphorylated vimentin may be elevated [1, 74, 77–79]. This elevated phosphorylation modulates the assembly of IFs by shifting the equilibrium constant of subunit exchange towards a higher dissociation rate [74]. Phosphorylation often causes conformational alterations by introducing negative charges, thereby altering the charge distribution and polarity of proteins. This modification may lead to structural rearrangements in vimentin, potentially disrupting the assembly and stability of filaments. The introduction of phosphate groups may also interfere with the binding affinity between IFs, reducing the stability of their interactions and facilitating their disassembly and solubility.

Phosphorylation is catalyzed by a broad range of protein kinases, such as PKC, CDK1, PAK, PKA, CaMKII, and ROCK. This PTM can lead to the disassembly of vimentin filaments, particularly during processes like cell division, migration and inflammation [1, 35, 80, 81]. In contrast, dephosphorylation can promote filament reassembly and stabilize the IF network. The balance between phosphorylation and dephosphorylation events may be essential for the dynamic regulation of vimentin filament assembly and disassembly to enable cell responses to various physiological and mechanical cues.

### Glutathionylation

For this PTM, the tripeptide glutathione is reversibly attached to cysteine residues in target proteins such as vimentin to form mixed disulfides. This process serves as a signaling mechanism for cellular responses to oxidative stress and inflammation [82]. Except for peripherin, which has two, all other type III IFs proteins contain a single cysteine residue. For vimentin, the highly conserved Cys328 is located within the terminal helical segment of the rod domain, which is located near the region where adjacent ULF domains interdigitate during filament elongation. As longitudinal assembly of ULFs is required for the formation of extended filaments, modifications to Cys328 could potentially lead to aberrant IF assembly. Consistent with this notion, S-glutathionylation of Cys328 blocks the longitudinal assembly of ULFs and may be important for enabling the IF network’s responsiveness to oxidative stress. Cys328 in vimentin is a target for oxidative and electrophilic modifications, which markedly influence vimentin structure and function [83].

### Sumoylation

Sumoylation is orchestrated by a cascade of enzymatic actions that typically target lysine residues (K) within the consensus sequences ψ-K-X-E or ψ-K-X-D of target proteins. This nomenclature denotes ψ as a large hydrophobic amino acid, X indicates any amino acid, and D and E indicate the acidic residues aspartic and glutamic acids, respectively [84]. This PTM attaches small ubiquitin-like modifiers (SUMO) covalently to the target protein, thereby influencing protein stability and intracellular distribution [85, 86]. Sumoylation is involved in regulating various biological functions of immune cells [87]. In the context of vimentin disassembly, a notable example is the role of PIAS1, an E3 SUMO ligase that is also a key regulator in the inflammatory cascade [88]. PIAS1 mediates sumoylation of vimentin at lysine residues within the canonical consensus motifs, Lys439 and Lys445. This modification is pivotal as it promotes the disassembly of vimentin filaments and modulates cell migration [89].

### Glycosylation

O-linked glycosylation of vimentin attaches N-acetylglucosamine molecules to specific serine or threonine amino acid residues in the head domain, including Ser34 and Ser39. While these residues are also potential phosphorylation sites, a priori glycosylation may block phosphorylation. Other glycosylation sites (not necessarily phosphorylation sites), such as Ser49, have also been investigated. Glycosylation of Ser49 residues within the two dimeric head domains structurally interconnects these domains, thereby promoting the assembly of vimentin dimers and likely, increased stabilization of filaments. Glycosylation of Ser49 is also involved in vimentin-dependent regulation of cell migration. Further, glycosylation is involved in bacterial replication after Chlamydia infection by inducing the inclusion of vimentin into cage-like structures that protect the bacteria [90].

### ADP-ribosylation

In this reversible modification, ADP-ribose units (derived from nicotinamide adenine dinucleotide (NAD+) are added to amino acid residues such as arginine. This process is catalyzed by ADP-ribosyltransferases and is associated with chronic inflammatory and infectious diseases [91]. For vimentin, ADP-ribosylation occurs primarily at Arg44 and Arg49 within the head domain, a critical region for the regulation of vimentin assembly. Similar to phosphorylation, ADP-ribosylation can inhibit the assembly of vimentin into filaments. Vimentin filaments are also ADP-ribosylated by bacterial Streptococcus pyogenes that express ADP-ribosyltransferase. Modification of arginine residues in the head domain inhibits vimentin filament formation, which facilitates IF network disassembly [92].

### Citrullination

Arginine is converted to citrulline in a reaction that is catalyzed by Peptidyl Arginine Deiminases (PADs). For this modification, NH is changed to an O in the guanidino group of arginine, which alters charge, conformation, and antigenic epitopes, thereby affecting its immunogenic properties. Citrullination can occur in certain physiological processes such as hair follicle formation [93]. In inflammatory environments, the level of citrullination may rise, possibly related to the Ca^2+^ dependence of PADs. Typically, in healthy cells, the intracellular concentration of Ca^2+^ is insufficient to trigger PADs activation. Nonetheless, in the context of inflamed tissues, such as the synovium of rheumatoid arthritis, PADs are activated through an influx of Ca^2+^, which then leads to vimentin citrullination [94]. As vimentin is enriched in arginine residues, it is particularly susceptible to citrullination by PADs. In rheumatoid arthritis, autoantibodies are produced against citrullinated protein antigens. Notably, citrullinated vimentin is one of the most highly cited candidate antigens linked to rheumatoid arthritis that trigger autoimmune reactions [95]. In addition to altering the potential antigenicity of epitopes in vimentin, citrullination facilitates the disassembly of vimentin into soluble forms that promote secretion as ECV [96].

### Acetylation

Acetylation involves the addition of an acetyl group to the lysine residue of a protein, which neutralizes the positive charge of the lysine. This modification has the potential to alter protein structure and function. Vimentin can undergo acetylation at Lys120, which regulates its stability and functionality [97]. While the precise impact of acetylation on vimentin structure is not well-defined, acetylation may reduce electrostatic interactions between vimentin molecules by neutralizing the positive charge of lysines. This reduction in electrostatic interactions may contribute to the disassembly of vimentin filaments.

### Ubiquitination

Ubiquitination regulates protein degradation, intracellular localization, and function by adding ubiquitin molecules to the lysine residues of proteins. It is known that vimentin can be regulated by ubiquitination, which affects its degradation and consequently influences cellular function [98].

When considering the overall impact of various PTMs on vimentin structure, while glycosylation favors the assembly of vimentin, all of the other PTMs discussed here enhance the disassembly of vimentin. Collectively these PTMs encourage the production of more soluble forms of vimentin that are subsequently released as ECV. Future research that defines how discrete PTMs are regulated by pro-inflammatory cytokines would be helpful in terms of linking ECV release to inflammation.

## The mechanism of vimentin externalization

**Fig. 4:**
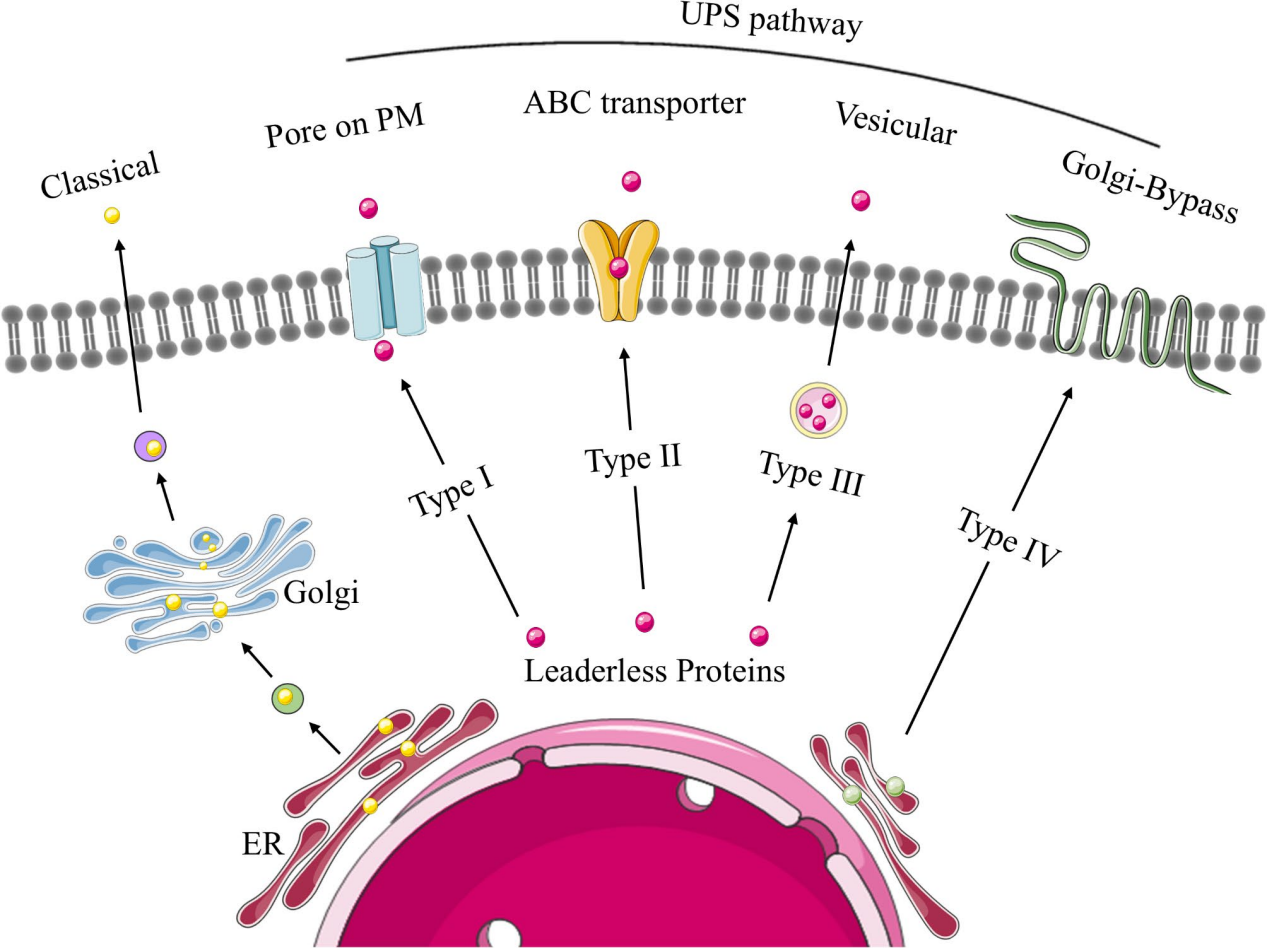
The classical protein secretory pathway (ER-Golgi secretory pathway) and four types of unconventional protein secretion (UPS) pathways

ECV has been associated with cell senescence and death because it was observed on the membrane of dying cells [99] and in tissues that exhibited loss of barrier function of the plasma membrane. Vimentin is actively externalized by macrophages after disassembly into soluble forms in response to inflammation [1] and is exposed on the surface of a subset of endothelial cells [100]. While no defined mechanisms for vimentin filament transport across the plasma membrane have been definitively established, several different types of vimentin secretion pathways have been considered, which we will review below. Conceivably, an improved understanding of vimentin secretion pathways will enhance our understanding of the diverse functions of ECV and the regulation of vimentin secretion in cellular and tissue pathology (Fig. 4).

The classical protein secretory pathway (i.e., the ER-Golgi secretory pathway) is one of the primary routes for protein transport and secretion. Briefly, nascent proteins utilize a signal peptide that directs them into the endoplasmic reticulum where they undergo PTMs and quality control [101]. Proteins that meet quality standards are packaged into vesicles coated with COPII proteins, transported to the Golgi apparatus [102] and subsequently packaged into secretory vesicles that bud off from the Golgi. These vesicles fuse with the plasma membrane, releasing their contents into the extracellular space through exocytosis. In this classical pathway, secreted proteins carry out their designated functions [103] but the features of ECV described above are not consistent with this pathway. ECV lacks a signal peptide [71], which is a general requirement for proteins to be recognized and directed to the ER for secretion.

Instead of the classical pathway described above, some proteins are released from cells through unconventional protein secretion (UPS) pathways. UPS pathways can be categorized into two major groups based on the presence or absence of a signal peptide in the secreted protein (Table 3). UPS pathways are usually triggered by cellular stress and inflammation, conditions that could impair the functionality of the classical secretory pathway. Notably, recent studies suggest that intracellular soluble vimentin is exported to the extracellular space via discrete UPS pathways [2].

**Table 3:** Protein secretion pathways of potential relevance to ECV

		Features	Secretion Pathway	Signal Peptide	Example proteins
**Classical Secretory Pathway**		Recognition of signal peptide	ER→ Golgi→ PM	Yes	Collagens
**UPS** **pathway**	**Type I**	Pore on the PM-mediated	Protein domains to form a channel into PM	No	FGF2[104], TAT[105]
	**Type II**	ABC transporter-based	Energy of ATP binding and hydrolysis	No	Acylated peptides, yeast mating peptides[106]
	**Type III**	Autophagosome/ endosome-based	Packaged into PM-bound organelles	No	Vimentin[2], IL-18[107], HMGB1[108], DKK3[109]
	**Type IV**	Bypass Golgi	ER→ PM	Yes	CFTR[110], CD45[111], Pendrin[112]

Proteins that are transported via the first three types of UPS pathways (Types 1-III) described in Table 3 lack a signal peptide and therefore cannot be recognized and directed to the ER. These three types of UPS pathways are described below:

### Type I UPS pathway

cytoplasmic proteins secreted through this pathway lack a signal peptide. They traverse the plasma membrane through self-assembled lipidic pores. Typical examples include Fibroblast Growth Factor 2 (FGF2) [104] and the HIV Trans-activator of Transcription (TAT) [105]. Vimentin cannot apparently be secreted through this pathway as there is no evidence of pore formation in the plasma membrane in cells that secrete vimentin.

### Type II pathway

involves ATP-binding cassette (ABC) transporter-based secretion. This pathway utilizes ATP-binding cassette (ABC) transporters and is dedicated to the secretion of acylated peptides and yeast mating peptides [106]. While not as deeply studied, this pathway is recognized as being distinct from other UPS pathways and as of yet no data have been obtained to indicate a role for this pathway in ECV production.

### Type III pathway

an autophagosome/endosome-based secretion pathway that relies on membrane-bound organelles such as endosomes and autophagosomes that are diverted from their normal functions to become secretory. Typically, cytoplasmic or nuclear secretory proteins are released through this UPS pathway under specific environmental conditions. These proteins often serve as mediators of the inflammatory response and in angiogenesis, cell growth and differentiation. For instance, the secretion of IL-18 [107], HMGB1 [108], DKK3 [109] is thought to be dependent on this pathway.

### Type IV pathway

Proteins that also contain a signal peptide and/or a transmembrane domain enter the ER but bypass the Golgi apparatus on their way to the plasma membrane (Golgi-Bypass Pathway, Type IV Pathway) [113]. This pathway is predominantly associated with transmembrane proteins rather than secretory proteins [114], like soluble vimentin. For instance, CFTR [110], CD45 [111], pendrin [112], which are transmembrane proteins, are conveyed directly from the ER to the plasma membrane, thereby bypassing the Golgi. These proteins can reach the plasma membrane even if transport from the ER to the Golgi is compromised.

In a recent study investigating the secretion mechanisms of vimentin by endothelial cells [2], the relative abundance of vimentin secretion correlated with endothelial cell activation: angiogenic factors promoted release while anti-angiogenic agents reduced release. Culture of cells with inhibitors of classical protein secretion mechanisms, which targeted the ER and Golgi apparatus, did not suppress release of vimentin. However, treatment of cells with inhibitors of autophagosome/lysosome/endosome-based secretion UPS pathway reduced vimentin secretion. Collectively these data indicate that at least for endothelial cells, vimentin is most likely secreted through a type III UPS pathway.

In astrocytes, after injury or stress (e.g., following central nervous system damage) the expression of vimentin is upregulated. Within the cell, vimentin is encapsulated into multivesicular bodies, which then fuse with the cell membrane to form extracellular vesicles, which are subsequently released into the extracellular space. Astrocytes employ this mechanism to release astroglial vimentin via extracellular vesicles [4], which can then bind the insulin-like growth factor 1on neurons, a process that could be pivotal for the CNS injury response, inflammation, and the progression of neurodegenerative diseases. Adipocyte progenitor cells can also release vimentin on the surface of exosomes to facility wound repair [3].

While the secretion of ECV in CNS and some other cell types appears to involve extracellular vesicles, this mechanism is not universally supported by studies of other cell types, such as immune cells or fibroblasts, in inflammatory contexts. Further research is needed to clarify these discrepancies and understand the broader implications for cellular communication and disease progression.

## ECV secretion in inflamed lesions and specific pathologies

In inflammatory lesions, ECV is primarily secreted by immune cells and by resident stromal cells like fibroblasts; this secretory process may be considered as a response to inflammation in sites to which immune cells are recruited. Inflammatory cells including monocytes-macrophages [1, 115], neutrophils [116, 117] and lymphocytes [18] release vimentin when activated or when undergoing apoptosis. For instance, in the inflammatory response to bacterial challenges exhibited by macrophages, vimentin secretion is induced and augmented by pro-inflammatory factors like TNF-α [1] and by oxidized low density lipoprotein [19]. In the context of tuberculosis infection, vimentin is secreted to the cell surface of monocytes and serves as a recognition pattern for natural killer cells, which facilitate their cytotoxicity against infected cells [118]. In neutrophil extracellular trap (NET) formation and activation (a process known as NETosis), PADs are activated, which leads to the citrullination of histones released from cells.

As vimentin is also a substrate for PADs enzymes, disassembly of vimentin filaments in the neutrophil cytoskeleton during NETosis allows the DNA liberates from heterochromatin to pass through the cell into the extracellular space [17]. Enhanced NETosis, which is characterized by the extrusion of citrullinated vimentin, also contributes to the aberrant adaptive and innate immune responses observed in the pathogenesis of rheumatoid arthritis [116]. Further, ECV release has been detected in instances of mechanical cell damage [119], senescence [99] and apoptosis [120]. In these various contexts, ECV is a common response to inflammation and tissue damage.

In healthy tissues in which immune cell infiltration is limited, ECV is found at low levels such as in the lung [39] and periodontium [121]. But in inflamed tissues, substantial numbers of immune cells are recruited and activated, which leads to increased ECV secretion. These pathologically increased levels of ECV are typically associated with systemic and/or local dysregulation of tissue structure and function, as observed in various inflammatory or neoplastic diseases (Fig. 5). Accordingly, ECV may serve as a biomarker for monitoring the status of inflammation and tissue health.

The concentrations of secreted or surface-bound ECV are increased in inflammatory and autoimmune disease or after injury. The plasma concentration of vimentin in patients experiencing critical illness with systemic inflammatory response is notably elevated, averaging a two-fold increase compared to that observed in healthy subjects [122]. Serum levels of vimentin are markedly increased in individuals diagnosed with coronary artery disease compared with healthy patients [19]. In atherosclerotic lesions, ECV is enriched in the core area of necrotic lesions and in sites with abundant numbers of activated inflammatory cells [123]. Muscle injury also leads to an increased expression of ECV [16].

The presence of ECV in the circulation likely contributes to the increased formation of vimentin antibodies, as observed in allograft rejection [124], syngeneic cardiac transplant rejection [125], pulmonary fibrosis [126], systemic lupus erythematosus [127], Behçet’s disease (BD) [128], and the generation of citrullinated vimentin in rheumatoid arthritis [129]. Moreover, the presence of vimentin antibodies correlates with the clinical manifestations or prognosis of these diseases [126, 129].

Excessive ECV influences various pathological processes, including inflammatory responses, fibrosis, and pathogen invasion. During inflammation, increased levels of ECV can serve as damage-associated molecular pattern and trigger inflammation-associated signaling cascades such as NF-κB [1, 19]. This activation establishes a self-perpetuating feedback loop that intensifies the inflammatory response, which could contribute to the progression of conditions like atherogenic inflammation and periodontitis [123, 130]. Moreover, vimentin transitions into soluble forms and is released extracellularly during injury. In this context, elevated ECV induces mesenchymal repair cells to differentiate into myofibroblasts, resulting in excessive extracellular matrix secretion and wound fibrosis [131]. Further, the presence of ECV on the surface of host cells may act as an attachment platform, thereby facilitating pathogen invasion and potentially exacerbating infection-related complications [16, 132]. Antibodies to vimentin have been identified as biomarkers for various diseases [133]. Specifically, certain antibodies may offer protection against inflammatory diseases [134] and cancer [135, 136], highlighting their potential role in diagnostics and therapy.

## Conclusions

- **Vimentin’s Roles and Functions**: vimentin is involved in a variety of physiological and pathological processes, including immune cell activation, inflammation, and tissue repair. In these situations, vimentin expression may be upregulated and vimentin molecules may be post-translationally modified to enable signaling events that extend beyond the cell type that originally expressed the vimentin.
- **Vimentin Assembly**,** Release**,** and PTMs**: The structure and function of intracellular vimentin can adapt to alterations of the extracellular environment by altering filament assembly and conformation. Some intracellular vimentin is modified through PTMs such as phosphorylation or citrullination, which lead to its release as oligomers into the extracellular space.
- **ECV Secretion Mechanism and Inflammatory Response**: ECV can be released through the type III UPS pathway, and its secretion is modulated by inflammatory cytokines.
- **ECV Secretion in Specific Pathologies**: The concentration of ECV and the production of vimentin antibodies in blood or bodily fluids increase under several different inflammatory and autoimmune-related conditions. Moreover, high levels of ECV contribute to various pathological processes, including inflammatory responses, fibrosis, and pathogen invasion, underscoring their potential significance in the diagnosis and treatment of inflammatory diseases.
- **ECV in infection**,** wound healing**,** inflammation**,** cancer**,** and fibrosis**: ECV or vimentin antibodies are often used as markers of pathology, such as in rheumatoid arthritis. ECV can facilitate wound healing under defined conditions, but sometimes ECV triggers pathological processes. For example, ECV can: (1) act as an adhesive co-receptor for bacterial and viral entry into host cells; (2) activate leader cells after wounding of the eye lens, potentially leading to fibrosis; (3) promote pro-fibrotic responses in injured lung by enhancing phagocytosis by macrophages; (4) trigger neutrophils through TLR4 to promote extravasation; (5) stimulate angiogenesis to promote tumor growth.

**Future research possibilities**

- Define the enzymatic systems that drive those PTMs which are required for conversion of vimentin filaments to oligomers and that are secreted as ECV.
- Use cultured cells and animal models to identify the specific inflammatory signaling pathways that regulate the generation of vimentin. These approaches would be useful first steps to assess whether ECV is a potential therapeutic target for diseases characterized by excessive inflammation or impaired tissue repair.
- Conduct human studies of inflammatory diseases such as rheumatoid arthritis or periodontitis to explore the diagnostic and therapeutic potential of ECV in respectively, blood samples or gingival crevicular fluid.

## Data Availability

No datasets were generated or analysed during the current study.
